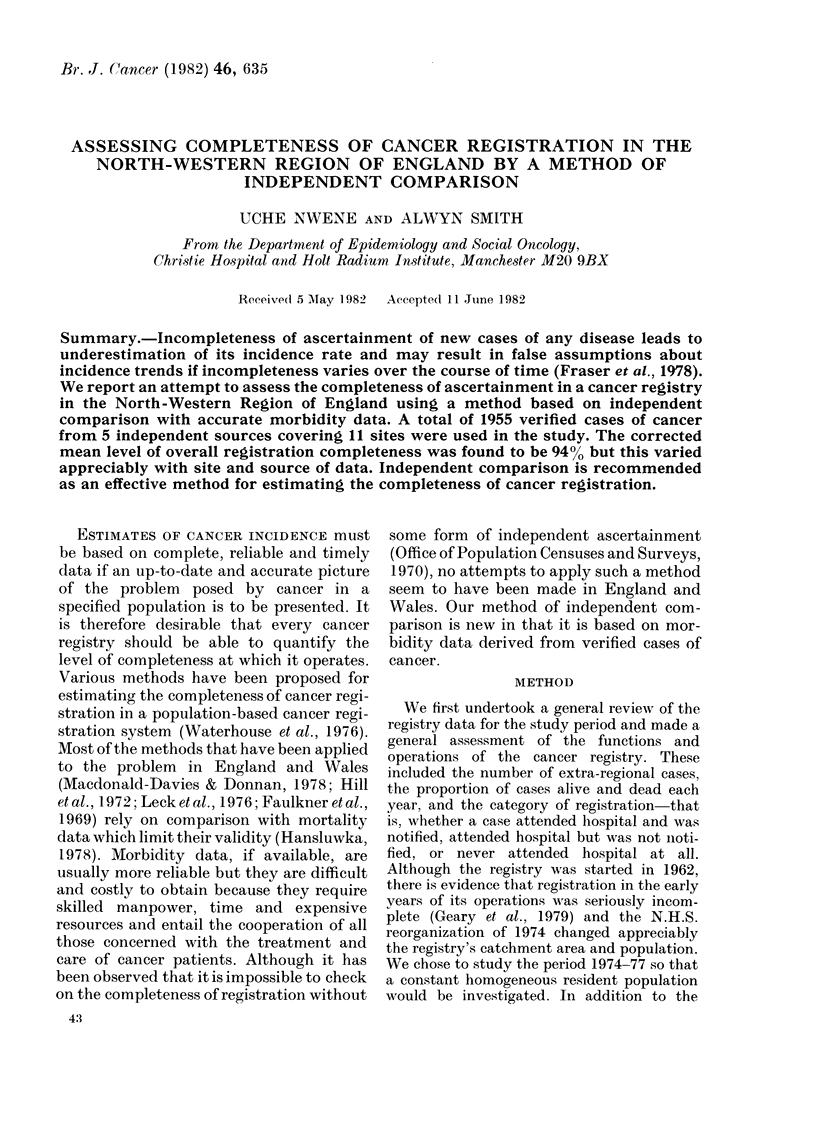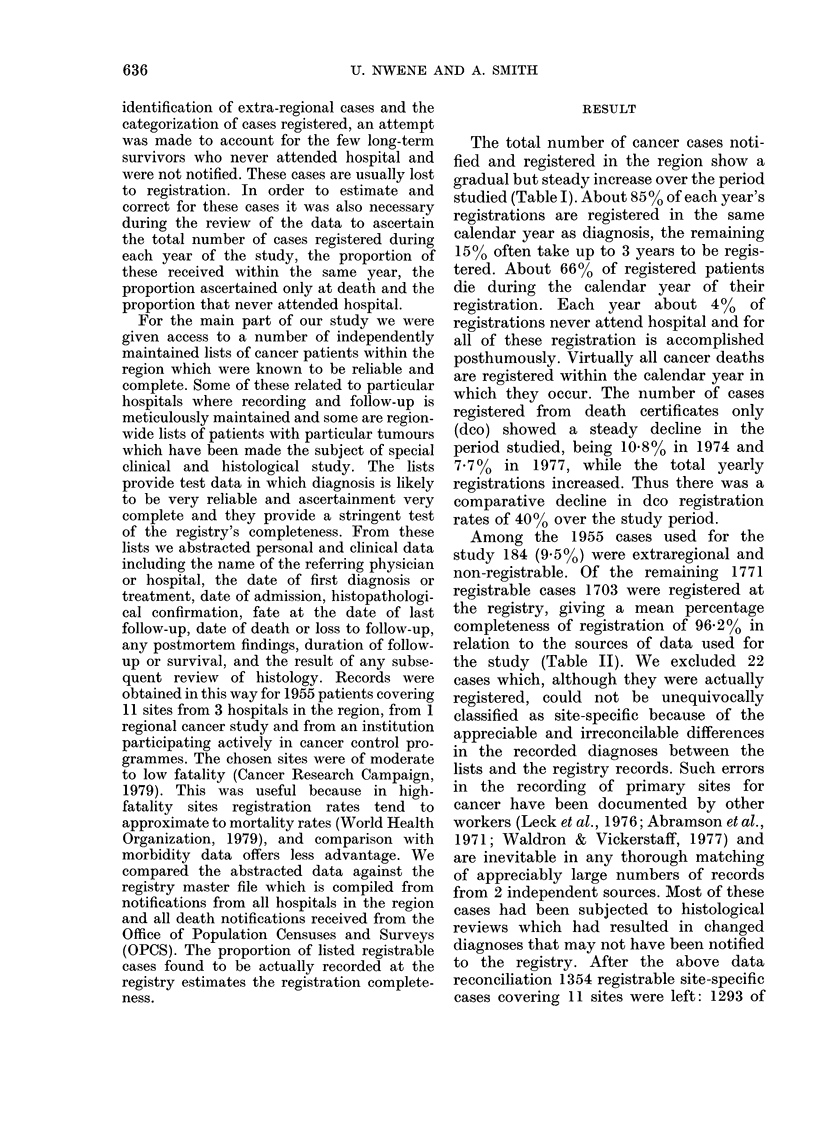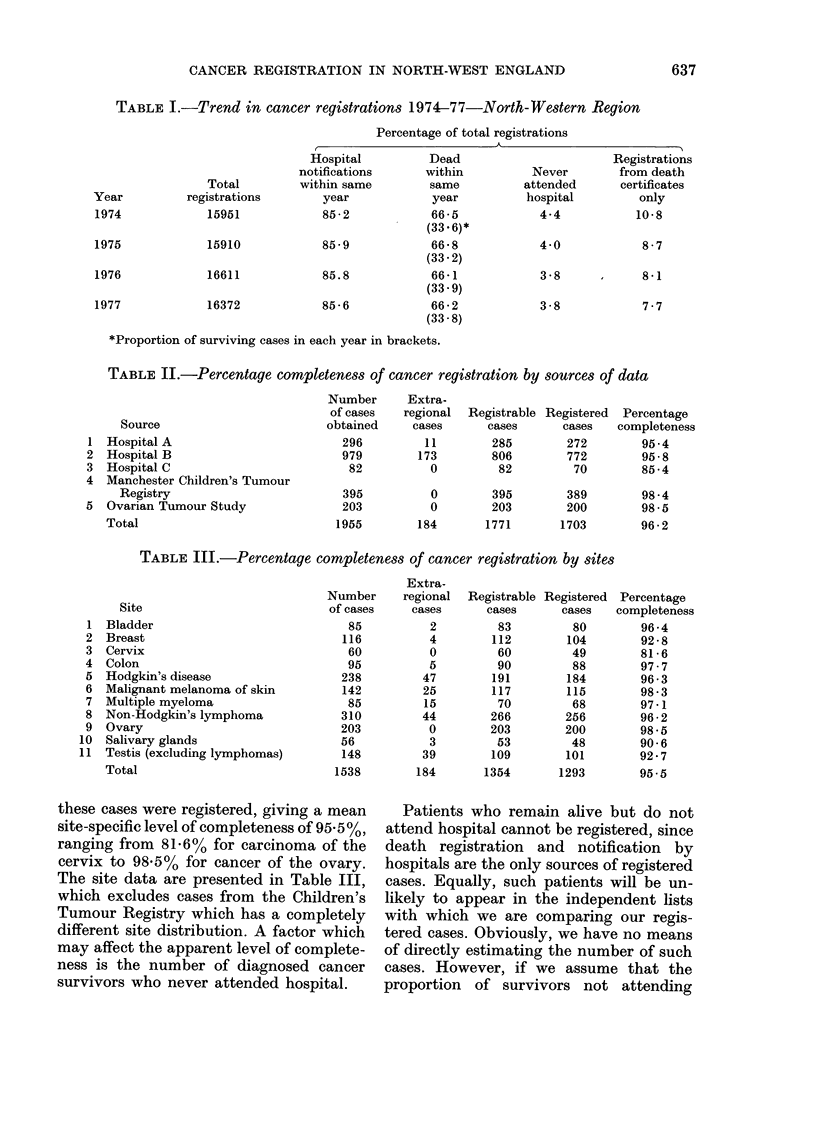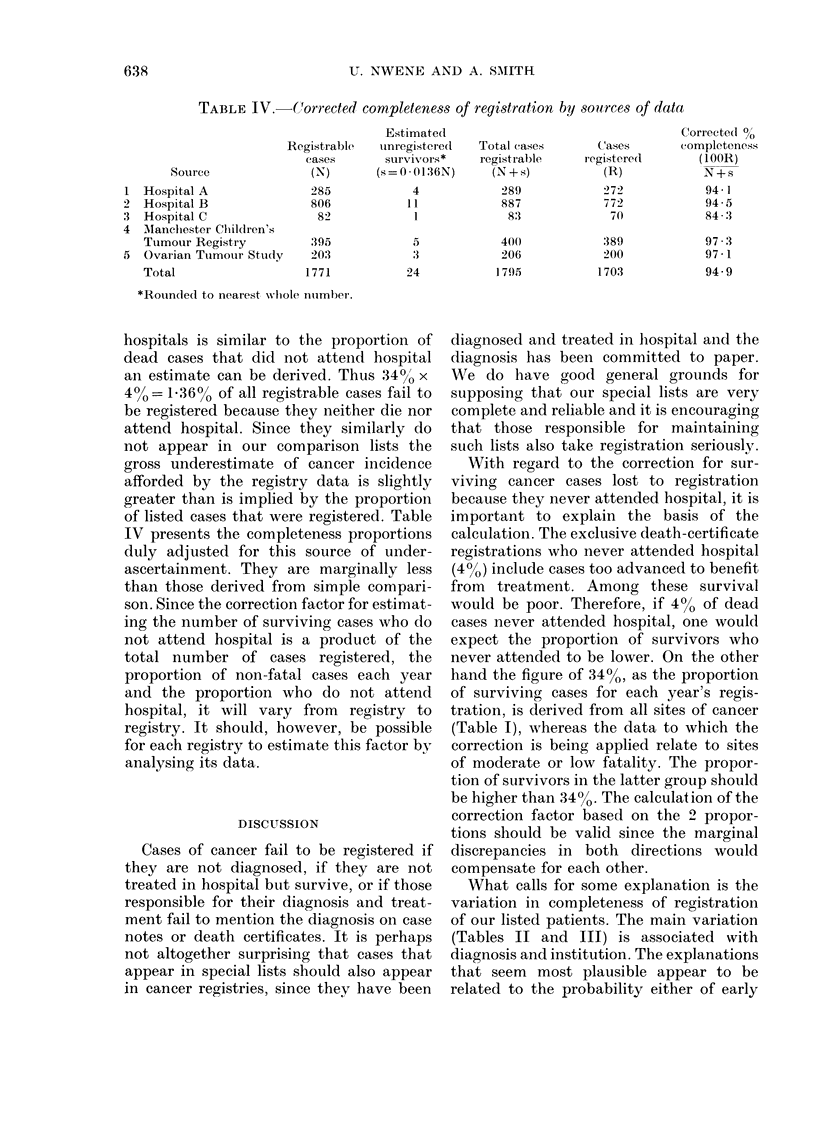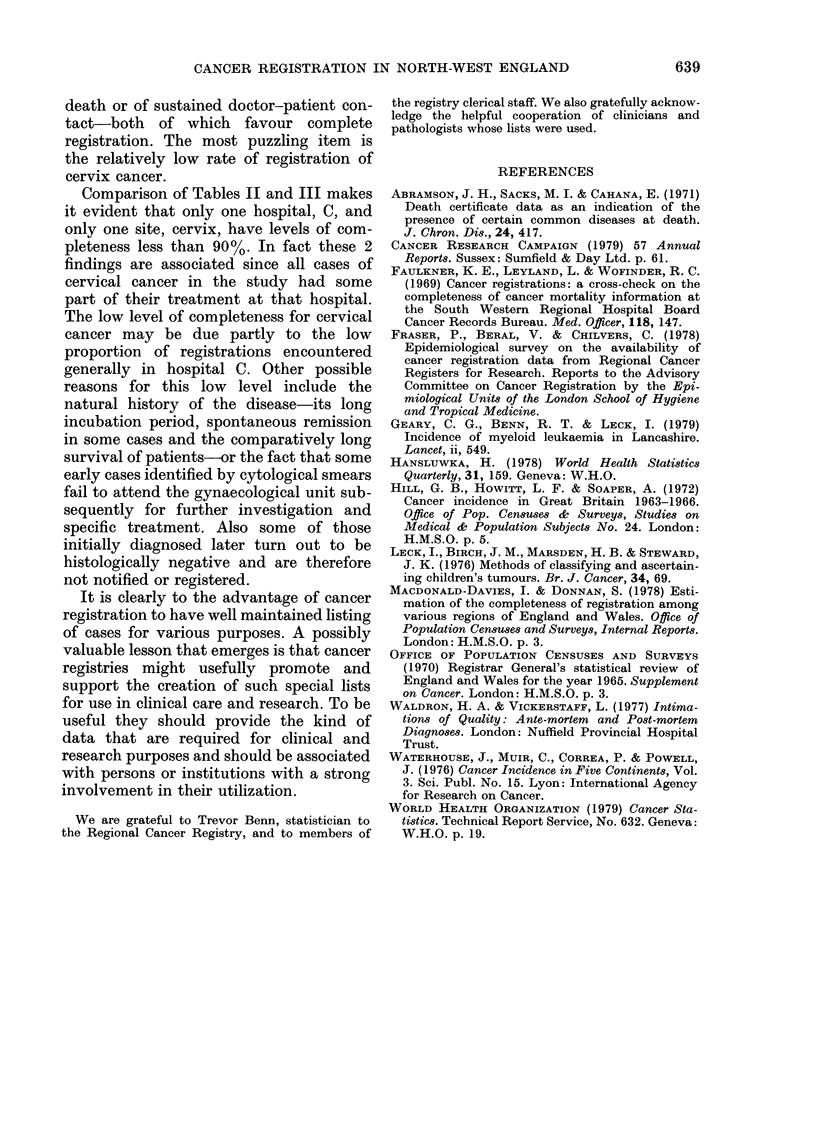# Assessing completeness of cancer registration in the north-western region of England by a method of independent comparison.

**DOI:** 10.1038/bjc.1982.248

**Published:** 1982-10

**Authors:** U. Nwene, A. Smith

## Abstract

Incompleteness of ascertainment of new cases of any disease leads to underestimation of its incidence rate and may result in false assumptions about incidence trends if incompleteness varies over the course of time (Fraser et al., 1978). We report an attempt to assess the completeness of ascertainment in a cancer registry in the North-Western Region of England using a method based on independent comparison with accurate morbidity data. A total of 1955 verified cases of cancer from 5 independent sources covering 11 sites were used in the study. The corrected mean level of overall registration completeness was found to be 94% but this varied appreciably with site and source of data. Independent comparison is recommended as an effective method for estimating the completeness of cancer registration.


*Br. J. Cancer* (1982) **46**, 635

### ASSESSING COMPLETENESS OF CANCER REGISTRATION IN THE NORTH-WESTERN REGION OF ENGLAND BY A METHOD OF INDEPENDENT COMPARISON

UCHE NWENE AND ALWYN SMITH

*From the Department of Epidemiology and Social Oncology, Christie Hospital and Holt Radium Institute, Manchester M20 9BX*

Received 5 May 1982 Accepted 11 June 1982

**Summary.—Incompleteness of ascertainment of new cases of any disease leads to underestimation of its incidence rate and may result in false assumptions about incidence trends if incompleteness varies over the course of time (Fraser *et al.*, 1978). We report an attempt to assess the completeness of ascertainment in a cancer registry in the North-Western Region of England using a method based on independent comparison with accurate morbidity data. A total of 1955 verified cases of cancer from 5 independent sources covering 11 sites were used in the study. The corrected mean level of overall registration completeness was found to be 94% but this varied appreciably with site and source of data. Independent comparison is recommended as an effective method for estimating the completeness of cancer registration.**

ESTIMATES OF CANCER INCIDENCE must be based on complete, reliable and timely data if an up-to-date and accurate picture of the problem posed by cancer in a specified population is to be presented. It is therefore desirable that every cancer registry should be able to quantify the level of completeness at which it operates. Various methods have been proposed for estimating the completeness of cancer registration in a population-based cancer registration system (Waterhouse *et al.*, 1976). Most of the methods that have been applied to the problem in England and Wales (Macdonald-Davies & Donnan, 1978; Hill *et al.*, 1972; Leck *et al.*, 1976; Faulkner *et al.*, 1969) rely on comparison with mortality data which limit their validity (Hansluwka, 1978). Morbidity data, if available, are usually more reliable but they are difficult and costly to obtain because they require skilled manpower, time and expensive resources and entail the cooperation of all those concerned with the treatment and care of cancer patients. Although it has been observed that it is impossible to check on the completeness of registration without some form of independent ascertainment (Office of Population Censuses and Surveys, 1970), no attempts to apply such a method seem to have been made in England and Wales. Our method of independent comparison is new in that it is based on morbidity data derived from verified cases of cancer.

### METHOD

We first undertook a general review of the registry data for the study period and made a general assessment of the functions and operations of the cancer registry. These included the number of extra-regional cases, the proportion of cases alive and dead each year, and the category of registration—that is, whether a case attended hospital and was notified, attended hospital but was not notified, or never attended hospital at all. Although the registry was started in 1962, there is evidence that registration in the early years of its operations was seriously incomplete (Geary *et al.*, 1979) and the N.H.S. reorganization of 1974 changed appreciably the registry's catchment area and population. We chose to study the period 1974–77 so that a constant homogeneous resident population would be investigated. In addition to the identification of extra-regional cases and the categorization of cases registered, an attempt was made to account for the few long-term survivors who never attended hospital and were not notified. These cases are usually lost to registration. In order to estimate and correct for these cases it was also necessary during the review of the data to ascertain the total number of cases registered during each year of the study, the proportion of these received within the same year, the proportion ascertained only at death and the proportion that never attended hospital.

For the main part of our study we were given access to a number of independently maintained lists of cancer patients within the region which were known to be reliable and complete. Some of these related to particular hospitals where recording and follow-up is meticulously maintained and some are region-wide lists of patients with particular tumours which have been made the subject of special clinical and histological study. The lists provide test data in which diagnosis is likely to be very reliable and ascertainment very complete and they provide a stringent test of the registry's completeness. From these lists we abstracted personal and clinical data including the name of the referring physician or hospital, the date of first diagnosis or treatment, date of admission, histopathological confirmation, fate at the date of last follow-up, date of death or loss to follow-up, any postmortem findings, duration of follow-up or survival, and the result of any subsequent review of histology. Records were obtained in this way for 1955 patients covering 11 sites from 3 hospitals in the region, from 1 regional cancer study and from an institution participating actively in cancer control programmes. The chosen sites were of moderate to low fatality (Cancer Research Campaign, 1979). This was useful because in high-fatality sites registration rates tend to approximate to mortality rates (World Health Organization, 1979), and comparison with morbidity data offers less advantage. We compared the abstracted data against the registry master file which is compiled from notifications from all hospitals in the region and all death notifications received from the Office of Population Censuses and Surveys (OPCS). The proportion of listed registrable cases found to be actually recorded at the registry estimates the registration completeness.

### RESULT

The total number of cancer cases notified and registered in the region show a gradual but steady increase over the period studied (Table I). About 85% of each year's registrations are registered in the same calendar year as diagnosis, the remaining 15% often take up to 3 years to be registered. About 66% of registered patients die during the calendar year of their registration. Each year about 4% of registrations never attend hospital and for all of these registration is accomplished posthumously. Virtually all cancer deaths are registered within the calendar year in which they occur. The number of cases registered from death certificates only (dco) showed a steady decline in the period studied, being 10·8% in 1974 and 7·7% in 1977, while the total yearly registrations increased. Thus there was a comparative decline in dco registration rates of 40% over the study period.

Among the 1955 cases used for the study 184 (9·5%) were extraregional and non-registrable. Of the remaining 1771 registrable cases 1703 were registered at the registry, giving a mean percentage completeness of registration of 96·2% in relation to the sources of data used for the study (Table II). We excluded 22 cases which, although they were actually registered, could not be unequivocally classified as site-specific because of the appreciable and irreconcilable differences in the recorded diagnoses between the lists and the registry records. Such errors in the recording of primary sites for cancer have been documented by other workers (Leck *et al.*, 1976; Abramson *et al.*, 1971; Waldron & Vickerstaff, 1977) and are inevitable in any thorough matching of appreciably large numbers of records from 2 independent sources. Most of these cases had been subjected to histological reviews which had resulted in changed diagnoses that may not have been notified to the registry. After the above data reconciliation 1354 registrable site-specific cases covering 11 sites were left: 1293 of

TABLE I.—*Trend in cancer registrations* 1974–77—*North-Western Region*

| Year | Total registrations | Percentage of total registrations: Hospital notifications within same year | Dead within same year | Never attended hospital | Registrations from death certificates only |
|---|---|---|---|---|---|
| 1974 | 15951 | 85·2 | 66·5 (33·6)* | 4·4 | 10·8 |
| 1975 | 15910 | 85·9 | 66·8 (33·2) | 4·0 | 8·7 |
| 1976 | 16611 | 85.8 | 66·1 (33·9) | 3·8 | 8·1 |
| 1977 | 16372 | 85·6 | 66·2 (33·8) | 3·8 | 7·7 |

*Proportion of surviving cases in each year in brackets.

TABLE II.—*Percentage completeness of cancer registration by sources of data*

| | Source | Number of cases obtained | Extra-regional cases | Registrable cases | Registered cases | Percentage completeness |
|---|---|---|---|---|---|---|
| 1 | Hospital A | 296 | 11 | 285 | 272 | 95·4 |
| 2 | Hospital B | 979 | 173 | 806 | 772 | 95·8 |
| 3 | Hospital C | 82 | 0 | 82 | 70 | 85·4 |
| 4 | Manchester Children's Tumour Registry | 395 | 0 | 395 | 389 | 98·4 |
| 5 | Ovarian Tumour Study | 203 | 0 | 203 | 200 | 98·5 |
| | Total | 1955 | 184 | 1771 | 1703 | 96·2 |

TABLE III.—*Percentage completeness of cancer registration by sites*

| | Site | Number of cases | Extra-regional cases | Registrable cases | Registered cases | Percentage completeness |
|---|---|---|---|---|---|---|
| 1 | Bladder | 85 | 2 | 83 | 80 | 96·4 |
| 2 | Breast | 116 | 4 | 112 | 104 | 92·8 |
| 3 | Cervix | 60 | 0 | 60 | 49 | 81·6 |
| 4 | Colon | 95 | 5 | 90 | 88 | 97·7 |
| 5 | Hodgkin's disease | 238 | 47 | 191 | 184 | 96·3 |
| 6 | Malignant melanoma of skin | 142 | 25 | 117 | 115 | 98·3 |
| 7 | Multiple myeloma | 85 | 15 | 70 | 68 | 97·1 |
| 8 | Non-Hodgkin's lymphoma | 310 | 44 | 266 | 256 | 96·2 |
| 9 | Ovary | 203 | 0 | 203 | 200 | 98·5 |
| 10 | Salivary glands | 56 | 3 | 53 | 48 | 90·6 |
| 11 | Testis (excluding lymphomas) | 148 | 39 | 109 | 101 | 92·7 |
| | Total | 1538 | 184 | 1354 | 1293 | 95·5 |

these cases were registered, giving a mean site-specific level of completeness of 95·5%, ranging from 81·6% for carcinoma of the cervix to 98·5% for cancer of the ovary. The site data are presented in Table III, which excludes cases from the Children's Tumour Registry which has a completely different site distribution. A factor which may affect the apparent level of completeness is the number of diagnosed cancer survivors who never attended hospital.

Patients who remain alive but do not attend hospital cannot be registered, since death registration and notification by hospitals are the only sources of registered cases. Equally, such patients will be unlikely to appear in the independent lists with which we are comparing our registered cases. Obviously, we have no means of directly estimating the number of such cases. However, if we assume that the proportion of survivors not attending

TABLE IV.—*Corrected completeness of registration by sources of data*

| Source | Registrable cases (N) | Estimated unregistered survivors* (s = 0·0136N) | Total cases registrable (N + s) | Cases registered (R) | Corrected % completeness (100R)/(N+s) |
|---|---|---|---|---|---|
| 1 Hospital A | 285 | 4 | 289 | 272 | 94·1 |
| 2 Hospital B | 806 | 11 | 887 | 772 | 94·5 |
| 3 Hospital C | 82 | 1 | 83 | 70 | 84·3 |
| 4 Manchester Children's Tumour Registry | 395 | 5 | 400 | 389 | 97·3 |
| 5 Ovarian Tumour Study | 203 | 3 | 206 | 200 | 97·1 |
| Total | 1771 | 24 | 1795 | 1703 | 94·9 |

*Rounded to nearest whole number.

hospitals is similar to the proportion of dead cases that did not attend hospital an estimate can be derived. Thus 34% × 4% = 1·36% of all registrable cases fail to be registered because they neither die nor attend hospital. Since they similarly do not appear in our comparison lists the gross underestimate of cancer incidence afforded by the registry data is slightly greater than is implied by the proportion of listed cases that were registered. Table IV presents the completeness proportions duly adjusted for this source of under-ascertainment. They are marginally less than those derived from simple comparison. Since the correction factor for estimating the number of surviving cases who do not attend hospital is a product of the total number of cases registered, the proportion of non-fatal cases each year and the proportion who do not attend hospital, it will vary from registry to registry. It should, however, be possible for each registry to estimate this factor by analysing its data.

### DISCUSSION

Cases of cancer fail to be registered if they are not diagnosed, if they are not treated in hospital but survive, or if those responsible for their diagnosis and treatment fail to mention the diagnosis on case notes or death certificates. It is perhaps not altogether surprising that cases that appear in special lists should also appear in cancer registries, since they have been diagnosed and treated in hospital and the diagnosis has been committed to paper. We do have good general grounds for supposing that our special lists are very complete and reliable and it is encouraging that those responsible for maintaining such lists also take registration seriously.

With regard to the correction for surviving cancer cases lost to registration because they never attended hospital, it is important to explain the basis of the calculation. The exclusive death-certificate registrations who never attended hospital (4%) include cases too advanced to benefit from treatment. Among these survival would be poor. Therefore, if 4% of dead cases never attended hospital, one would expect the proportion of survivors who never attended to be lower. On the other hand the figure of 34%, as the proportion of surviving cases for each year's registration, is derived from all sites of cancer (Table I), whereas the data to which the correction is being applied relate to sites of moderate or low fatality. The proportion of survivors in the latter group should be higher than 34%. The calculation of the correction factor based on the 2 proportions should be valid since the marginal discrepancies in both directions would compensate for each other.

What calls for some explanation is the variation in completeness of registration of our listed patients. The main variation (Tables II and III) is associated with diagnosis and institution. The explanations that seem most plausible appear to be related to the probability either of early death or of sustained doctor–patient contact—both of which favour complete registration. The most puzzling item is the relatively low rate of registration of cervix cancer.

Comparison of Tables II and III makes it evident that only one hospital, C, and only one site, cervix, have levels of completeness less than 90%. In fact these 2 findings are associated since all cases of cervical cancer in the study had some part of their treatment at that hospital. The low level of completeness for cervical cancer may be due partly to the low proportion of registrations encountered generally in hospital C. Other possible reasons for this low level include the natural history of the disease—its long incubation period, spontaneous remission in some cases and the comparatively long survival of patients—or the fact that some early cases identified by cytological smears fail to attend the gynaecological unit subsequently for further investigation and specific treatment. Also some of those initially diagnosed later turn out to be histologically negative and are therefore not notified or registered.

It is clearly to the advantage of cancer registration to have well maintained listing of cases for various purposes. A possibly valuable lesson that emerges is that cancer registries might usefully promote and support the creation of such special lists for use in clinical care and research. To be useful they should provide the kind of data that are required for clinical and research purposes and should be associated with persons or institutions with a strong involvement in their utilization.

We are grateful to Trevor Benn, statistician to the Regional Cancer Registry, and to members of the registry clerical staff. We also gratefully acknowledge the helpful cooperation of clinicians and pathologists whose lists were used.

### REFERENCES

ABRAMSON, J. H., SACKS, M. I. & CAHANA, E. (1971) Death certificate data as an indication of the presence of certain common diseases at death. *J. Chron. Dis.*, **24**, 417.

CANCER RESEARCH CAMPAIGN (1979) 57 *Annual Reports.* Sussex: Sumfield & Day Ltd. p. 61.

FAULKNER, K. E., LEYLAND, L. & WOFINDER, R. C. (1969) Cancer registrations: a cross-check on the completeness of cancer mortality information at the South Western Regional Hospital Board Cancer Records Bureau. *Med. Officer*, **118**, 147.

FRASER, P., BERAL, V. & CHILVERS, C. (1978) Epidemiological survey on the availability of cancer registration data from Regional Cancer Registers for Research. Reports to the Advisory Committee on Cancer Registration by the *Epimiological Units of the London School of Hygiene and Tropical Medicine.*

GEARY, C. G., BENN, R. T. & LECK, I. (1979) Incidence of myeloid leukaemia in Lancashire. *Lancet*, ii, 549.

HANSLUWKA, H. (1978) *World Health Statistics Quarterly*, **31**, 159. Geneva: W.H.O.

HILL, G. B., HOWITT, L. F. & SOAPER, A. (1972) Cancer incidence in Great Britain 1963–1966. *Office of Pop. Censuses & Surveys, Studies on Medical & Population Subjects No.* 24. London: H.M.S.O. p. 5.

LECK, I., BIRCH, J. M., MARSDEN, H. B. & STEWARD, J. K. (1976) Methods of classifying and ascertaining children's tumours. *Br. J. Cancer*, **34**, 69.

MACDONALD-DAVIES, I. & DONNAN, S. (1978) Estimation of the completeness of registration among various regions of England and Wales. *Office of Population Censuses and Surveys, Internal Reports.* London: H.M.S.O. p. 3.

OFFICE OF POPULATION CENSUSES AND SURVEYS (1970) Registrar General's statistical review of England and Wales for the year 1965. *Supplement on Cancer.* London: H.M.S.O. p. 3.

WALDRON, H. A. & VICKERSTAFF, L. (1977) *Intimations of Quality: Ante-mortem and Post-mortem Diagnoses.* London: Nuffield Provincial Hospital Trust.

WATERHOUSE, J., MUIR, C., CORREA, P. & POWELL, J. (1976) *Cancer Incidence in Five Continents*, Vol. 3. Sci. Publ. No. 15. Lyon: International Agency for Research on Cancer.

WORLD HEALTH ORGANIZATION (1979) *Cancer Statistics.* Technical Report Service, No. 632. Geneva: W.H.O. p. 19.